# Sleep and Cognition in the Long Life Family Study

**DOI:** 10.1002/alz.089972

**Published:** 2025-01-09

**Authors:** Emma Gordon, Yian Gu, Stephanie Cosentino, Stacy L. Andersen, Svetlana Ukraintseva

**Affiliations:** ^1^ Albert Einstein College of Medicine, Bronx, NY USA; ^2^ Columbia University Irving Medical Center, New York, NY USA; ^3^ Boston University School of Medicine, Boston, MA USA; ^4^ Duke University, Durham, NC USA

## Abstract

**Background:**

Many studies have demonstrated an association between sleep and cognition in older adults. The Long Life Family Study (LLFS) has followed families enriched with longevity since 2006 and has shown that this cohort has slower rates of cognitive decline than their spouse controls and the general public. Understanding how sleep and cognition are related in this population can improve our understanding of the key to long life.

**Method:**

Our study includes 2344 participants from the LLFS, who were ≥60 years at baseline, and completed sleep and cognitive measures. A neuropsychological battery was used to measure episodic (Logical Memory IA and IIA from the Wechsler Memory Scale ‐Revised (WMS‐R)), semantic (Categorical Fluency Test), and working memory (Number Span Forward and Backward Tests) at baseline (2006‐2009), and in a subset of 1633 participants at the follow‐up visits 6.9 (SD = 2.7) years later. Sleep habits were assessed once using the Health Habits (HH) Questionnaire between the two cognitive measures. The association between sleep duration and memory was examined using generalized estimating equations models that accounted for relationship with the long‐life families.

**Result:**

Compared to those with 6.5‐8.5 hours of sleep, those with > 8.5 hours of sleep had lower episodic memory scores at baseline (b = ‐0.705, p = 0.021), but not at follow‐up (b = ‐0.627, p = 0.149), after controlling for age, sex, field center, APOE, and generation. Sleep duration was not associated with semantic or working memory. Those who had > 8.5 hours of sleep had faster decline in all memory scores, although not significantly. There was no difference in memory scores comparing < 6.5 hours of sleep to 6.5‐8.5 hours of sleep.

**Conclusion:**

Lower memory scores at baseline predicted longer sleep time subsequently, while sleep time did not predict memory performance on follow‐up. Overall, the current study did not support sleep duration as a significant contributor to memory decline. This contradicts some earlier findings and prompts further investigation into the connection between sleep and cognition, especially within this population and specifically into whether increased sleep time and memory decline share an underlying mechanism, or memory decline causes longer sleep time.

